# Editorial

**DOI:** 10.1108/MIJ-11-2019-008

**Published:** 2019-11-04

**Authors:** Kevin Wei, Emnet Alemu

**Affiliations:** University of California Irvine, Irvine, California, USA

## Editorial commentary on “nursing students and depressive symptomatology. an observational study in University of Palermo”

Santangelo *et al.* (2019) identify the socio-demographic correlates of depressive symptomatology amongst nursing students in the hopes of raising public awareness. The nursing student population faces unique stressors. Compared to regular students, they are likely to become anxious about future job placement, work–life balance, medical errors and patient death (Chen *et al.*, 2019; Fernandes *et al.*, 2018). The study raises an important question about the relationship between nursing education and depression.

According to the authors, being in a relationship is a protective risk factor against depression. It promotes feelings of interpersonal connectedness, thereby reduces depressive symptomatology. Similarly, marriage is shown to be a protective factor in the general population. However, depression tends to make a person socially withdraw and become isolated. Therefore, it might not be such a strong protective factor.

In the study, physical illness is a risk factor for depression symptoms. Nursing students who suffer from physical illness were likely to report depressive symptoms. The finding is plausible, as chronic illness relates to depression in the standard population. Interestingly, untreated depression may manifest with somatic symptoms. Therefore, some nursing students might be wrongly attributing psychosomatic symptoms to a physical ailment.

Additionally, the second and third years of nursing school were linked positively to depressive symptoms. There are several reasons to explain relationship. With higher levels of education, there might be increased workload and responsibilities. Also, it could be related to worrying more about post-graduation job placement. Further studies are needed to clarify the exact relationship between the year of study and depression symptomatology. It would be helpful to create a prospective cohort study to better establish a connection with depressive symptoms and year of study.

Santangelo *et al.* do a great job raising awareness of the risk factors for depression in the nursing population. Future research should include multiple university centers to determine if the findings are similar. Examining a broader variety of nursing programs would make these results more generalizable. It would also help establish guidelines to address discrepancies in mental health. In addition, studies should focus on decreasing risk factors for depressive symptomatology among nursing students. Future studies could look into the effectiveness of university implemented wellness programs. Wellness programs could reduce risk factors for depression by improving the students’ social support network and by encouraging them to lead a healthy lifestyle. For instance, school-organized wellness programs could offer yoga classes, nutritional cooking sessions and peer support groups.
